# Simplifying Antibiotic Management of Peritonitis in APD: Evidence from a Non-Inferiority Randomized Trial

**DOI:** 10.3390/antibiotics14080747

**Published:** 2025-07-24

**Authors:** Jesús Venegas-Ramírez, Benjamín Trujillo-Hernández, Carmen Citlalli Castillón-Flores, Fernanda Janine Landín-Herrera, Erika Herrera-Oliva, Patricia Calvo-Soto, Rosa Tapia-Vargas, Alejandro Figueroa-Gutiérrez, Eder Fernando Ríos-Bracamontes, Karina Esmeralda Espinoza-Mejía, Iris Anecxi Jiménez-Vieyra, Luis Antonio Bermúdez-Aceves, Blanca Judith Ávila-Flores, Efrén Murillo-Zamora

**Affiliations:** 1 Coordinación Auxiliar Médica de Investigación en Salud, Jefatura de Servicios de Prestaciones Médicas, Instituto Mexicano del Seguro Social, Colima 28030, Mexico; 2 Facultad de Medicina, Universidad de Colima, Colima 28040, Mexico; 3 Hospital General de Zona No. 1, Instituto Mexicano del Seguro Social, Villa de Álvarez 28984, Mexico; 4 Facultad de Medicina, Universidad Autónoma de Guadalajara, Zapopan 44100, Mexico; 5 Unidad de Medicina Familiar No. 3, Instituto Mexicano del Seguro Social, Armería 28300, Mexico; 6 Coordinación de Planeación y Enlace Institucional, Jefatura de Servicios de Prestaciones Médicas, Instituto Mexicano del Seguro Social, Colima 28030, Mexico; 7 Unidad de Medicina Familiar No. 19, Instituto Mexicano del Seguro Social, Colima 28000, Mexico; 8 Unidad de Investigación en Epidemiología Clínica, Instituto Mexicano del Seguro Social, Villa de Álvarez 28984, Mexico

**Keywords:** automated peritoneal dialysis, peritonitis, peritoneal dialysis, continuous ambulatory, controlled clinical trials, randomized

## Abstract

Introduction/Objective: Peritonitis remains a serious complication in patients undergoing automated peritoneal dialysis (APD), requiring prompt and effective antibiotic administration. This study evaluated whether delivering antibiotics directly through APD bags is as effective as administering them via an additional manual daytime exchange. Methods: We conducted a randomized, single-blind, non-inferiority clinical trial involving patients diagnosed with peritonitis. Participants were randomly assigned to receive Ceftazidime and Vancomycin, either via APD bags or through a combined approach of continuous ambulatory peritoneal dialysis (CAPD) plus APD. A total of 64 patients (32 per group) were enrolled, with comparable baseline demographic and clinical profiles, including laboratory markers of infection severity and dialysis history. Results: Peritonitis resolved in 90.6% of the patients treated via APD bags and in 81.3% of those receiving antibiotics through manual exchange plus APD. Although this difference did not reach statistical significance (p = 0.281), the observed absolute difference of 9.3% was well within the predefined non-inferiority margin of 30%, supporting the clinical non-inferiority of the APD-only method. The mean time to resolution was similar between groups (p = 0.593). Post hoc power analyses indicated limited statistical power (18.5% for the resolution rate and 9.2% for time to resolution), suggesting that modest differences may not have been detectable given the sample size. Nevertheless, the high resolution rates observed in both groups reflect valid and encouraging clinical outcomes. Conclusion: Antibiotic administration via APD bags demonstrated comparable clinical effectiveness to the combined manual exchange plus APD method for treating peritonitis. Given its operational simplicity and favorable results, the APD-only strategy may offer a pragmatic alternative in routine care. Further studies with larger sample sizes are recommended to confirm these findings and optimize treatment protocols. Trial registration: NCT04077996. Funding source: None to declare.

## 1. Introduction

Peritonitis remains a life-threatening complication in patients receiving long-term automated peritoneal dialysis (APD), contributing significantly to both morbidity and mortality. Immediate clinical intervention is essential, as recent estimates report mortality rates of 3.1 and 5.7 per 1000 person-months for patients on APD and continuous ambulatory peritoneal dialysis (CAPD), respectively [1,2].

Intraperitoneal antibiotic therapy is the mainstay of treatment, as recommended by the International Society for Peritoneal Dialysis. However, despite growing concerns about potential under-dosing of antibiotics in APD, there is currently no consensus on the optimal method of antibiotic delivery in this context [3]. A recent study highlighted marked practice variation in antibiotic administration among clinicians in Australia and New Zealand, underscoring the need for standardized, evidence-based approaches [4].

The timely and appropriate administration of antibiotics is critical to ensure infection resolution and prevent long-term complications. Nevertheless, several aspects of antibiotic administration in APD, including the mode of delivery and dwell time, remain areas of uncertainty and clinical variability [5].

One commonly used strategy is a temporary switch to CAPD, which allows for more frequent and sustained antibiotic contact with the peritoneal cavity via four manual exchanges per day [6,7]. While potentially effective, this approach imposes logistical burdens, including the need for additional training of patients, caregivers, and nursing personnel, as well as increased healthcare costs and delays in treatment initiation [8].

An alternative method involves supplementing APD with one additional daytime manual exchange containing antibiotics. This approach may reduce the frequency of dosing while maintaining drug exposure in the peritoneal cavity during a 6 h dwell period, followed by the regular APD regimen [9]. However, it may also increase costs due to greater consumption of dialysis fluid and supplies, and it may require additional caregiver support and patient training [10].

A third approach entails the direct administration of antibiotics into APD cycler bags, distributing the total dose across the nighttime exchanges. Prior studies suggest that key antibiotics, such as Ceftazidime, remain stable in Icodextrin and glucose-based solutions for up to 24 h at clinically relevant concentrations and temperatures. When co-administered with Vancomycin, Ceftazidime has demonstrated stability rates exceeding 90% over a 24 h period in both 1.4% and 3.9% glucose solutions [11,12].

Despite these available options, comparative data on the clinical effectiveness of antibiotic administration via APD bags versus a supplemental daytime manual exchange combined with APD remain limited [13]. Generating such evidence is essential to improve the management of peritonitis in APD and to guide the development of standardized protocols. This study aimed to compare the effectiveness of two antibiotic administration strategies for the treatment of peritonitis in long-term APD patients: (1) intraperitoneal antibiotic administration via APD cycler bags and (2) administration through an additional daytime manual exchange combined with APD.

## 2. Results

Data from 64 patients were included in the analysis, as 2 patients in the intervention group were lost to follow-up because of the voluntary abandonment of dialytic therapy. Therefore, each study arm comprised 32 patients.

In the analysis of the etiological profile, *Enterobacter cloacae*, *Staphylococcus epidermidis*, and *Staphylococcus aureus* were the most frequently isolated pathogens. Although no significant differences were observed for most microorganisms between the study groups, *Staphylococcus aureus* was identified more frequently in the manual exchange plus APD group than in the APD bags group (31.3% vs. 9.4%, p = 0.027) (Table 1).

The characteristics of the participants, stratified by the randomly assigned groups, are presented in Table 1. No significant differences were observed between the study arms in terms of laboratory parameters, demographic data, or any of the 17 evaluated comorbid conditions. Additionally, the profile of peritoneal dialysis use, including treatment length and modality, was found to be homogeneous between the study arms.

The prevalence of a previous peritonitis episode was 62.5% (*n* = 20/32) in the intervention group and 53.1% (*n* = 17/32) in the control group, with no statistically significant difference (*p* = 0.448). None of the analyzed individuals received tidal peritoneal dialysis (TPD).

Finally, peritonitis resolution was documented in 90.6% (n = 29/32; 95% confidence interval [CI] 75.0–98.0%) of the patients who received antibiotics through APD bags and in 81.3% (n = 26/32; 95% CI 63.6–92.8%) of those who received them via manual exchange/day and APD. These proportions were statistically similar (p = 0.281). The post hoc power analysis indicated that this study had limited statistical power (18.5%) to detect a difference of this magnitude at α = 0.05. Non-inferiority analysis for the APD group was established with a priori margin of 30%, and the difference result was 9.3% (95% CI −15.4% to 16.4%).

The average number of days from the commencement of antibiotic treatment to the full resolution of peritonitis was 6.2 ± 2.9 and 5.7 ± 3.3 days for both the intervention and control groups. The post hoc power analysis indicated low statistical power (9.2%) to detect a difference of this magnitude.

## 3. Discussion

Our study aimed to compare the effectiveness of two treatment approaches in resolving peritonitis in long-term APD patients. The results showed comparable rates of clinical and paraclinical resolution of peritonitis in both treatment groups, indicating that both approaches are equally effective in achieving resolution and preventing further complications associated with the infection, for example, a change in modality to hemodialysis, catheter removal, or death.

To the best of our knowledge, there are no similar published studies that have directly compared the effectiveness of the administration of antibiotics in APD vs. the administration of antibiotics using a manual exchange/day plus APD approach, making our clinical trial unique in this regard. However, a study from a single center in Kuwait described a 10-year experience with peritonitis treatment and reported resolution rates of 65% for APD and 63% for CAPD patients [14]. Additionally, a Brazilian study documented a 2.5-fold increased risk of unresolved infection in peritonitis patients who switched from APD to CAPD [15]. While we did not find a statistically significant difference in resolution rates between the two groups in our study sample (81.3% vs. 90.6%, p = 0.281), the lower resolution rate in the control group (manual exchange/day plus APD) could be partially attributed to insufficient retraining in the use of manual dialysis and potential hydric overload due to its use.

Ensuring the stability of antibiotics, such as Ceftazidime and Vancomycin, in PD liquid is crucial for their efficacy and safety in treating peritonitis in PD patients. Prior studies have investigated the behavior of these antibiotics when introduced into the peritoneal cavity, and both Ceftazidime and Vancomycin have shown favorable stability profiles in PD liquid, with minimal degradation over time [15]. This preservation of antimicrobial activity is vital for achieving adequate plasma concentrations above the minimum inhibitory requirements, effectively combating bacterial infections in PD patients. In our study, both treatment groups received Ceftazidime and Vancomycin, which are the recommended empirical agents for PD patients with peritonitis [16].

Despite the valuable findings, our study has certain limitations that should be acknowledged. Firstly, the lack of double blinding for medical personnel and patients was necessary because this study was conducted within hospitals and involved a large group of non-blinded doctors and nurses. Second, the relatively small sample size (n = 64) may limit the statistical power to detect subtle differences between groups and restrict the generalizability of the findings to broader APD populations. Third, the short follow-up period of 15 days, while appropriate for assessing acute resolution of peritonitis, precludes evaluation of long-term outcomes, such as recurrence, catheter survival, or modality change.

Additionally, serum drug concentrations and intraperitoneal antibiotic levels were not measured, which limits pharmacokinetic comparisons between treatment groups. Although prior studies have demonstrated that minimum inhibitory concentrations for susceptible organisms are typically achieved when administering antibiotics via APD bags [17], direct measurement of antibiotic concentrations in peritoneal fluid would have strengthened the mechanistic interpretation of our results. Furthermore, the impact of antibiotic regimens on patient diuresis could not be assessed because of incomplete fluid balance records.

Finally, although the quantification of parathyroid hormones (to diagnostic secondary hyperparathyroidism), ferritin, and the percentage of transferrin saturation levels is mandated by current clinical guidelines in public healthcare settings in Mexico [18], data regarding the levels of parathyroid hormones and ferritin percentage of saturation were only available for approximately 20% of the enrolled patients because of administrative reasons. These limitations provide opportunities for future research and improvements in the management of peritonitis in APD patients.

## 4. Materials and Methods

### 4.1. Study Design

**Figure 1:**
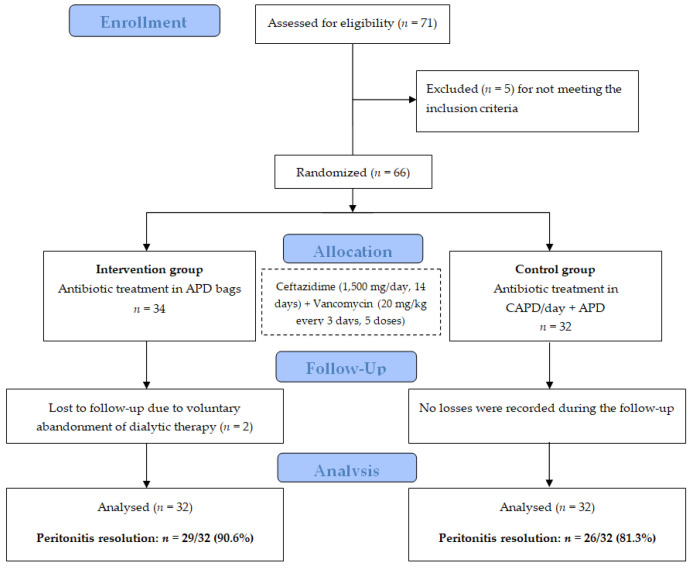
Study profile, Mexico, 2020–2022. Abbreviations: APD, automated peritoneal dialysis (APD); CAPD, continuous ambulatory peritoneal dialysis. Notes: (1) Peritonitis was defined by clinical symptoms (abdominal pain, fever, nausea, or vomiting), as well as laboratory criteria in the cytological examination of peritoneal fluid, which included a leucocyte count >100/µL and ≥50% polymorphonuclear cells, along with a positive Gram stain or bacterial culture. (2) The antibiotics were administered as follows: in the intervention group, the total antibiotic doses were divided and administered in a similar proportion to each APD bag, and in the control group, the antibiotic doses were applied in an extra manual daytime exchange with a 6 h dwell time inside the peritoneal cavity plus APD. (3) Participants were followed on a daily basis for 15 days. (4) The resolution of peritonitis was defined as the complete absence of clinical and laboratory data associated with peritonitis.

The study design adhered to the Standard Protocol Items: Recommendations for Interventional Trials (SPIRIT) [19] and Consolidated Standards of Reporting Trials (CONSORT) 2010 guidelines [20]. This research was a randomized, two-arm, parallel, single-blind, non-inferiority, multicenter interventional clinical trial. This study was conducted at 3 second-level public hospitals affiliated with the Mexican Institute of Social Security (*IMSS*, the Spanish acronym). All the hospitals are situated in west-central Mexico, specifically in the state of Colima, along the Pacific coast. Figure 1 summarizes the study design.

### 4.2. Participants

The inclusion criteria considered adult patients (aged 18 years or older) undergoing long-term APD in any of the study settings from January 2020 to January 2022, and for at least 3 months prior to their inclusion in this study. The eligible participants had to possess a fully functional catheter and meet the clinical criteria (abdominal pain, fever, nausea, or vomiting), as well as the laboratory criteria of peritonitis in the cytological exam of peritoneal fluid, which included a leucocyte count >100/µL and ≥50% polymorphonuclear cells, or with a positive Gram stain or bacterial culture [18]. Patients who were receiving any antibiotic at the onset of peritonitis, those with a history of allergy to Vancomycin or Ceftazidime, and those deemed unsuitable for peritoneal dialysis due to conditions such as abdominal hernias, abdominal wall abscess, or intra-abdominal collections were excluded from this study.

The following were the elimination criteria: adverse effects related to the antibiotic management of peritonitis (such as anaphylaxis, presence of Stevens–Johnson syndrome, toxic epidermal necrolysis, pseudomembranous colitis), intestinal perforation, dysfunction of the peritoneal dialysis catheter during this study, and patients who expressed their willingness to leave this study. Patients in whom the symptoms and laboratory data of peritonitis persisted after 5 days from the first dose of antibiotics were considered as cases of medically refractory peritonitis and were transitioned to hemodialysis. Patients who did not receive the totality of the programmed doses of antibiotics were excluded.

All the enrolled patients underwent training by three specialized and standardized nurses regarding the techniques involved in the administration of the evaluated antibiotics. During each hospital entry or scheduled medical appointment, the nursing staff evaluated the technique to ensure proper antibiotic administration.

### 4.3. Sample Size

The number of required participants was computed using formulas to compare two proportions, as commonly employed in non-inferiority clinical trials [21,22]. The calculation was based on the cure rate of peritonitis (75%) from a previously published study conducted in Mexico [23]. We opted for a conservative estimate of the proportion of cases that would experience the resolution of symptoms after receiving antibiotic treatment in APD bags (45%), thereby accounting for a potential 30% difference between the groups. By employing these formulas, a sample size of 30 patients per group (1:1 ratio) was determined to ensure a confidence interval of 95% and a statistical power of 80%. In this study, data from 64 patients (32 in each study arm) were analyzed.

### 4.4. Data Collection

Clinical data of interest were extracted from the medical records of the enrolled patients. Peritonitis-related symptoms were assessed through direct and guided interviews with closed questions conducted by three standardized physicians (one in each participating healthcare setting).

### 4.5. Intervention

Following the non-inferiority design of this trial and considering the widespread practice in our hospitals of administering antibiotics through extra manual daytime exchange in conjunction with APD for peritonitis cases, the study intervention involved antibiotic administration through APD bags, while the control group received the standard (an extra manual daytime exchange plus APD) approach. The participants were randomly assigned to one of the study arms using a table of random numbers. The randomization was carried out by an individual external to the research team (a social worker from one of the units where this study took place, who was unaware of the purpose of this research).

Both groups were provided with Ceftazidime at a dosage of 1500 mg per day for a duration of 14 days, coupled with Vancomycin at a dosage of 20 mg/kg every 3 days, resulting in a total of 5 doses. In the intervention group, the total dose was divided between both APD bags and remained in the cavity for the entire duration, in line with the prescribed regimen for each APD patient. The intermittent dose was allocated in an extra manual daytime exchange to the control group. In this context, we are referring to the dose being applied through an additional manual exchange during the day. The dwell time in the cavity of the supplementary bag in manual dialysis was 6 h, once a day, and during the night, APD was performed without antibiotics.

In most enrolled patients, dialysis bags containing 1.5% or 2.5% dextrose were utilized (intervention group, 91% of the patients; control group, 100%), with a median total volume of 10.6 ± 1.3 L and 10.4 ± 2.0 L in the intervention and control groups, respectively (p = 0.599).

### 4.6. Outcome

The main binary outcome (yes/no) of this study was the clinical and paraclinical resolution of peritonitis. It was defined as the complete disappearance of clinical symptoms (abdominal pain, fever, nausea, or vomiting) reported by the participants, along with the following characteristics observed in the peritoneal fluid from peritoneal exchange after 2 to 4 h of dwell time inside the cavity: clear macroscopic appearance, leucocyte count <100/µL, and < 50% of polymorphonuclear cells. Patients who did not meet all these criteria were considered negative for the analyzed outcome. The progression of peritonitis was assessed daily, and the evaluation continued until the disappearance of leucocytes and up to 15 days from the commencement of antibiotic administration.

### 4.7. Laboratory Methods

We utilized the Olympus AU480 Chemistry Analyzer (Beckman Coulter; Pasadena, CA, USA) to conduct the cytochemical analysis of peritoneal fluid samples. The microbiological procedures were conducted using the DxM MicroScan WalkAway ID/AST System (Beckman Coulter; Pasadena, CA, USA). This equipment is in each of the participating healthcare settings.

All culture media, including chocolate agar plates, were subjected to performance verification prior to inoculation using American Type Culture Collection (ATCC) reference strains to confirm viability and sterility [24]. The Gram staining procedures employed validated reagents with routine parallel processing of known positive and negative bacterial controls to ensure diagnostic accuracy. Technicians implemented daily equipment calibration logs and participated in external proficiency assessments [25]. These measures were designed to guarantee the reproducibility and reliability of microbiological findings across study sites.

During sample processing for bacterial culture, 50 mL of peritoneal fluid was centrifuged, and the supernatant was decanted. The sediment was then used for Gram staining and inoculation onto chocolate agar plates.

### 4.8. Statistical Analysis

Summary statistics were computed, and the significance level (α) was set at 5%. The statistical significance of proportions or means was assessed using chi-squared or *t*-tests, as appropriate. The analysis was conducted by one of the researchers who was kept uninformed about the intervention administered to the enrolled patients, as the data were presented with designations of either group A or B, devoid of any explicit particulars.

To support the interpretation of non-significant results, a post hoc power analysis was conducted following the primary outcome evaluation. This procedure estimated the likelihood of detecting clinically meaningful differences between groups given the observed effect sizes, sample sizes, and alpha level. Power calculations were performed using R software (4.4.2; R project, Vienna, Austria), applying standard assumptions for two-proportion and two-sample comparisons.

### 4.9. Ethical Considerations

This study was conducted according to the guidelines of the Declaration of Helsinki. Written informed consent to participate in this study was obtained from all the patients analyzed. This study was reviewed and approved by the National Committee of Ethics in Health Research (CNIC, the Spanish acronym) of the IMSS (approval number R-2019-785-005; 15 February 2020). The study protocol was registered with the National Library of Medicine of the US Government (Clinical trials.gov ID NCT04077996). No external funding was provided for the execution of this investigation.

## 5. Conclusions

This randomized trial provides clinically relevant evidence supporting the non-inferiority of administering intraperitoneal antibiotics exclusively via APD bags, compared to the combined use of a daytime manual exchange and APD, in the management of peritonitis among patients on long-term automated peritoneal dialysis. Both treatment strategies yielded high resolution rates, and the observed absolute difference remained well within the predefined non-inferiority margin. These findings reinforce the flexibility of APD-based antibiotic delivery and may facilitate streamlined care decisions in hospital or home-based settings, based on individual patient needs and logistical considerations. Further studies with larger sample sizes and extended follow-up are warranted to confirm these results and explore long-term outcomes.

## Figures and Tables

**Table 1:** Characteristics of the study groups for selected variables, Mexico, 2020–2022.

Characteristic	Intervention Group	Control Group	p
	APD Bags	Manual Exchange/Day +APD	
Age (years)	47.4 ± 14.6	51.5 ± 16.9	0.285
Gender			
Female	10 (31.3)	7 (21.9)	0.571
Male	22 (68.7)	25 (78.1)	
Body Mass Index	26.6 ± 4.9	27.9 ± 6.0	0.676
Erythropoietin, weekly dose (IU)	10,843 ± 5548	9437 ± 5593	0.194
PD, length of (months)	36.0 ± 32.9	30.3 ± 24.8	0.747
APD modality			
CCPD	29 (90.6)	30 (93.8)	0.641
INPD	3 (9.4)	2 (6.3)	
APD per day, duration (hours)	9.5 ± 0.6	9.6 ± 0.5	0.972
Exchanges per day (number)	5.2 ± 0.8	5.2 ± 0.9	0.954
C-reactive protein (mg/L)	112.1 ± 45.3	118.3 ± 47.9	0.642
Leukocyte count in blood (×10^9^/L)	11.4 ± 3.2	11.8 ± 3.5	0.684
Leucocyte count in dialysis fluid (×10^9^/L)	2050 ± 720	2180 ± 760	0.538
PMN in dialysis fluid (%)	84.5 ± 6.2	85.1 ± 5.9	0.712
Serum creatinine (mg/dL)	9.2 ± 2.4	9.0 ± 2.6	0.743
Isolated pathogen			
*Staphylococcus aureus*	3 (9.4)	10 (31.3)	0.027
*Enterobacter cloacae*	6 (18.8)	6 (18.8)	1.000
*Staphylococcus epidermidis*	6 (18.8)	1 (3.1)	0.107
*Pseudomonas aeruginosa*	2 (6.3)	3 (9.4)	0.640
*Escherichia colli*	2 (6.3)	2 (6.3)	1.000
*Klebsiella pneumoniae*	2 (6.3)	1 (3.1)	0.554
*Citrobacter freundii*	1 (3.1)	1 (3.1)	1.000
*Serratia marcescens*	1 (3.1)	0 (0)	0.313
*Achromobacter xylosoxidans*	1 (3.1)	0 (0)	0.313
*Candida* spp.	1 (3.1)	0 (0)	0.313
Other	7 (21.9)	8 (25.0)	0.774
*Personal history of*			
Tobacco use (yes)	18 (56.3)	20 (62.5)	0.611
Alcohol consumption (yes)	22 (68.8)	24 (75.0)	0.578
Type 2 diabetes mellitus (yes)	15 (46.9)	21 (65.6)	0.131
Diabetic foot (yes)	4 (12.5)	8 (25.0)	0.200
Diabetic retinopathy	10 (31.3)	12 (37.5)	0.599
Arterial hypertension (yes)	31 (96.9)	31 (96.9)	1.000
Ischemic heart disease (yes)	3 (9.4)	4 (12.5)	0.120
Stroke (yes)	3 (9.4)	1 (3.1)	0.302
Venous thrombosis (yes)	2 (6.3)	1 (3.1)	0.554
Hypertriglyceridemia (yes)	19 (55.9)	15 (46.9)	0.316
Hypercholesterolemia (yes)	16 (50.0)	16 (50.0)	1.000
Hyperuricemia (yes)	9 (28.1)	8 (25.0)	0.777
Cataract (yes)	16 (50.0)	11 (34.4)	0.206
Anemia (yes)	29 (90.6)	29 (90.6)	1.000
Catheter insertion site infection (yes)	8 (25.0)	6 (18.8)	0.545
Previous peritonitis episode (yes)	20 (62.5)	17 (53.1)	0.448

Abbreviations: APD, automated peritoneal dialysis; IUs, international units; PD, peritoneal dialysis; CCPD, continuous cycling peritoneal dialysis; INPD, intermittent nocturnal peritoneal dialysis; PMN, polymorphonuclear leukocyte. Note: (1) In the intervention group, antibiotics were administered in APD bags; in the control group, they were administered daily through continuous ambulatory peritoneal dialysis in conjunction with APD; (2) The *p*-values from ji-squared, fisher exact, or *t*-tests are presented, as appropriate.

## Data Availability

The data that support the findings of this study are available on request from the corresponding author.

## Abbreviations

The following abbreviations are used in this manuscript:

APD	Automated Peritoneal Dialysis
IMSS	Mexican Institute of Social Security (Spanish acronym)
CAPD	Continuous Ambulatory Peritoneal Dialysis
CCPD	Continuous Cycling Peritoneal Dialysis
CNIC	National Committee of Ethics in Health Research (Spanish acronym)
CONSORT	Consolidated Standards of Reporting Trials
SPIRIT	Recommendations for Interventional Trials
